# STRATEGIES FOR INTEGRATING ANTIRACIST PEDAGOGY INTO GERONTOLOGICAL EDUCATION

**DOI:** 10.1093/geroni/igac059.864

**Published:** 2022-12-20

**Authors:** Rona Karasik, Kyoko Kishimoto

**Affiliations:** St. Cloud State University, St. Cloud, Minnesota, United States; St. Cloud State University, St. Cloud, Minnesota, United States

## Abstract

Talking about diversity in gerontology is not new. Documenting racial disparities among older adults is not new. So what is different about applying antiracist pedagogy to teaching about aging and why should gerontology educators embrace it? Anti-racist pedagogy seeks to move beyond simply incorporating racial content into gerontology coursework. It involves acknowledging structural racism’s existence and relation to aging, uncovering its root causes in historical and political context, and studying its impact not just over the life course, but from generation to generation. Anti-racist pedagogy challenges students to learn how racial disparities among older adults emerged, why they persist, and what can be done about them. Applying anti-racist pedagogy can also challenge gerontological educators, who must find/prepare appropriate materials, as well as engage students in work that is potentially unfamiliar and/or uncomfortable. This presentation offers some strategies, examples, and pitfalls to avoid when incorporating anti-racist pedagogy into the gerontological curriculum.

